# Enhancing D-lactic acid production from methane through metabolic engineering of *Methylomonas* sp. DH-1

**DOI:** 10.1186/s12934-025-02695-z

**Published:** 2025-03-25

**Authors:** Seungwoo Cha, Jae-Hwan Jo, Jong Kwan Lee, Wooyoung Park, Myounghoon Moon, Gwon Woo Park, Min-Sik Kim, Ji-Sook Hahn

**Affiliations:** 1 Department of Chemical and Biological Engineering, Institute of Chemical Processes, Seoul National University, 1 Gwanak-Ro, Gwanak-Gu, Seoul, 08826 Republic of Korea; 2 Bioenergy and Resources Upcycling Research Laboratory, Korea Institute of Energy Research, 152 Gajeong-Ro, Yuseong-Gu, Daejeon, 34129 Republic of Korea; 3 Interdisciplinary Program for Agriculture and Life Sciences, Chonnam National University, 77 Yongbong-Ro, Buk-Gu, Gwangju, 61186 Republic of Korea; 4 Gwangju Clean Energy Research Center, Korea Institute of Energy Research, 270 Samso-Ro, Buk-Gu, Gwangju, 61003 Republic of Korea

**Keywords:** ADP-glucose, D-LA, Glucose-1-phosphate adenylyltransferase, Inducible promoter, Metabolic engineering, Methane, *Methylomonas* sp. DH-1

## Abstract

**Background:**

Methane is an abundant and low-cost carbon source with great potential for conversion into value-added chemicals. Methanotrophs, microorganisms that utilize methane as their sole carbon and energy source, present a promising platform for biotechnological applications. This study aimed to engineer *Methylomonas* sp. DH-1 to enhance D-LA production through metabolic pathway optimization during large-scale cultivation.

**Results:**

In this study, we regulated the expression of D-lactate dehydrogenase (D-LDH) using a P*tac* promoter with IPTG induction to mitigate the toxic effects of lactate accumulation. To further optimize carbon flow away from glycogen, the *glgA* gene was deleted. However, this modification led to growth inhibition, especially during scale-up, likely due to the accumulation of ADP-glucose caused by the rewired carbon flux under carbon-excess conditions. Deleting the *glgC* gene, which encodes glucose 1-phosphate adenylyltransferase, alleviated this issue. The final optimized strain, JHM805, achieved a D-LA production of 6.17 g/L in a 5-L bioreactor, with a productivity of 0.057 g/L/h, marking a significant improvement in D-LA production from methane.

**Conclusions:**

The metabolic engineering strategies employed in this study, including the use of an inducible promoter and alleviation of ADP-glucose accumulation toxicity, successfully enhanced the ability of the strain to produce D-LA from methane. Furthermore, optimizing the bioreactor fermentation process through methane and nitrate supplementation resulted in a significant increase in both the titer and productivity, exceeding previously reported values.

**Supplementary Information:**

The online version contains supplementary material available at 10.1186/s12934-025-02695-z.

## Background

Methane, a cost-effective and abundant carbon feedstock, is readily available for various natural and industrial processes. Advanced technologies, such as hydraulic fracturing and horizontal drilling, have improved methane extraction from shale gas, enhancing its use as a renewable feedstock for producing various value-added chemicals. [1–4]. Additionally, as the main component of biogas, methane can be sustainably derived from biomass, agricultural waste, and other organic materials, offering an eco-friendly option for biochemical and industrial applications [5, 6]. Given methane’s higher greenhouse gas potential compared to CO_2_, its utilization can also contribute to mitigating global warming.

The biological conversion of methane offers several advantages, including operation under mild conditions and absence of toxic by-products. Methanotrophic bacteria are promising biocatalysts because they utilize methane as their sole energy and carbon source. Significant efforts have been made to develop efficient genetic manipulation tools for these organisms. These tools include conjugation and electroporation methods for gene deletion and foreign DNA introduction [7–9], and negative selection systems such as *sacB* and mutant *pheS* counter-selection methods for multiple gene deletions [10, 11]. Additionally, marker-free chromosomal editing using the CRISPR/Cas9 system has been successfully implemented in both type I methanotroph *Methylococcus capsulatus* Bath and type II methanotroph *Methylocystis parvus* OBBP [12].

With the development of efficient genetic manipulation tools, methanotrophs have been increasingly explored in recent years to produce valuable chemicals from methane [13]. The chemicals produced from methane range from simple low-carbon compounds, such as methanol and acetate [14–16] to value-added medium-carbon chemicals, such as ectoine and cadaverine [17, 18]. More complex substances, such as terpenoids, fatty acids [19, 20], polyhydroxyalkanoates (PHA), and biodegradable thermoplastic polyesters [21–24], can also be produced. However, the titers of these products remain low compared to the results of conventional sugar fermentation through metabolic engineering of model microorganisms, such as *Escherichia coli* and *Saccharomyces cerevisiae*. The challenges of producing valuable compounds using engineered methanotrophic bacteria extend beyond the lack of information; they also include the genetic instability of strains when exposed to toxic chemicals. These limitations make the scaling up of bioprocesses for industrial applications particularly challenging. Many studies on fermenter-scale chemical production continue to report low productivity. In particular, few studies have successfully achieved gram-scale production of *Methylomonas* sp. DH-1 under various culture conditions [25–29], thereby highlighting the limitations of this methanotroph.

Organic acids, such as muconic acid, lactic acid, and 3-hydroxypropionic acid, can be used as building blocks for bioplastics [25, 30, 31]. With growing concerns over the increasing use of petroleum-based plastics, studies on bioplastic production have recently gained significant attention. In particular, poly-lactic acid (PLA), derived from lactic acid (LA), has attracted significant attention as a major bioplastic. Several approaches have been explored to produce LA from methane using type I methanotrophs, but the titer and yield have been limited by the toxicity of lactate within cells. In *Methylomicrobium buryatense* 5GB1S, the lactate dehydrogenase (*LDH*) gene was expressed on an episomal plasmid, resulting in 0.8 g/L of LA production in a continuous stirred bioreactor [32]. This titer matched the maximum LA tolerance of the strain, suggesting that intracellular accumulation of LA imposes a strict limitation on the production levels. Subsequent studies in the same strain reported enhancements in *LDH* expression through the engineering of promoters and ribosome binding sites [33]. Furthermore, in *Methylomicrobium alcaliphilum* 20z^R^, genes encoding the pyruvate dehydrogenase complex were deleted to increase pyruvate flux towards LA instead of acetyl-CoA. However, this manipulation failed to improve the titer, indicating that tolerance to LA is the primary bottleneck in LA production [34]. The challenge of LA production stems from the inherent toxicity of weak organic acids in microbial cells. When accumulated intracellularly, LA dissociates into lactate and protons, disrupting cellular homeostasis and normal function. This toxicity makes LA production using methanotrophs highly challenging, emphasizing the need for well-defined strategies to overcome these limitations.

In our previous study, we addressed this problem by performing adaptive laboratory evolution of *Methylomonas* sp. DH-1, resulting in JHM80, a strain exhibiting tolerance to 8.0 g/L of LA. We expressed the D-specific *LDH* gene from *Leuconostoc mesenteroides* (*Lm*.*LDH*) using a *glgBA* promoter while deleting the *glgA* gene, which encodes glycogen synthase to prevent carbon flux towards glycogen biosynthesis. As a result, this engineered strain produced 1.19 g/L of D-LA [25]. In this study, we used an inducible promoter to regulate the expression of *D*-*LDH* in JHM80. Furthermore, we found that deletion of the *glgA* gene inadvertently led to cell toxicity due to ADP-glucose accumulation. We addressed this growth inhibition by eliminating the *glgC* gene and optimizing the large-scale fermentation process, ultimately achieving record-high production of 6.17 g/L of D-LA in a 5-L fermenter.

## Methods

### Strains and culture conditions

All strains used in this study are listed in Table 1. Strains derived from *Methylomonas* sp. DH-1 were cultured in nitrate mineral salts (NMS) medium (0.49 g/L MgSO_4_, 1.0 g/L KNO_3_, 0.23 g/L CaCl_2_·2H_2_O, 3.8 mg/L Fe-EDTA, 0.5 mg/L Na_2_MoO_4_, 10 μM CuSO_4_·5H_2_O, with the addition of 1000X trace element solution, 100X vitamin stock and 100X phosphate stock solution). Cells were grown in 12.5 mL NMS medium in a 125 mL baffled flask or 50 mL NMS medium in a 500 mL baffled flask with rubber-type screw cap supplemented with 20% (v/v) methane and 80% air at 30 °C with shaking at 170 rpm. Proper concentrations of anhydrotetracycline (aTc) or IPTG were added to the medium for induction of *D-LDH* with 10 μg/mL of kanamycin. All growth and metabolic measurements were performed in biological duplicates unless otherwise specified.

**Table 1:** Strains used in this study

Strain	Genotype	References
*Methylomonas* sp. DH-1	Wild-type strain	[54]
JHM80	LA evolved strain from DH-1	[25]
JHM801	JHM80 *ΔglgA::Kan*^*R*^	This study
JHM802	JHM80 *ΔglgABC::Kan*^*R*^	This study
JHM803	JHM80 *ΔglgA::*P_*tetA*_*-tetR-*T_*rrnB*_*-*P_*tet*_*-Lm.LDH*-T_*rrnB*_*-Kan*^*R*^	This study
JHM804	JHM80 *ΔglgA::*P_*lacI*_-*lacI*-T_*rrnB*_*-*P_*tac*_*-Lm.LDH-*T_*rrnB*_*-Kan*^*R*^	This study
JHM805	JHM804 *ΔglgC::Amp*^*R*^	This study

### Plasmid construction and genetic manipulation of *Methylomonas* sp. DH-1

Plasmids used in this study to generate strains with chromosomal modifications are listed in Table 2. To construct plasmids for expression of lactate dehydrogenase from *L. mesenteroides* (*Lm.LDH*) with inducible promoters, we used pDel2-glgA-Lm.LDH as a parental plasmid which contains *Lm.LDH* flanked by 1-kb upstream (U_*glgA*_) and 1-kb downstream (D_*glgA*_) of *glgA* [25]. To provide restriction enzyme sites for promoter cloning, pDel3-glgA-Lm.LDH that contains *Mau*BI/*Bam*HI sites in front of the *Lm.LDH* gene was generated by using AccuRapid™ Cloning Kit (Bioneer, Korea). The P_*tet*_ and P_*tac*_ were cloned between *Mau*BI and *Bam*HI sites, resulting in pDel3-glgA-P_*tet*_-Lm.LDH and pDel3-glgA-P_*tac*_-Lm.LDH respectively. To construct plasmids for gene deletion, previously generated pDel2-fliE plasmid was used [25]. The 1-kb upstream and downstream sequences of target genes were amplified by PCR and cloned into *Not*I/*Spe*I and *Apa*I/*Sac*I sites in pDel2-fliE, respectively.

**Table 2:** Plasmids used in this study

Plasmid	Description	References
pDel2-glgA-Lm.LDH	pCM184-U_*glgA*_-[*Lm.LDH*-T_*rrnB*_-*Kan*^*R*^]-D_*glgA*_, without *Amp*^*R*^	[25]
pDel3-glgA-Lm.LDH	pDel2-glgA-Lm.LDH, containing *Mau*BI/*Bam*HI site for promoter cloning	This study
pDel3-glgA-P_*tet*_-Lm.LDH	U_*glgA*_-[P_tetA_-*tetR*-T_*rrnB*_-P_*tet*_-*Lm.LDH*-T_*rrnB*_-*Kan*^*R*^]-D_*glgA*_	This study
pDel3-glgA-P_*tac*_-Lm.LDH	U_*glgA*_-[P_*lacI*_-*lacI*-T_*rrnB*_-P_*tac*_-*Lm.LDH*-T_*rrnB*_-*Kan*^*R*^]-D_*glgA*_	This study
pDel2-filE	Plasmid containing [U_*fliE*_-T_*rrnB*_-*Kan*^*R*^-D_*fliE*_] cassette for *fliE* gene deletion	[25]
pDel2-glgA	pDel2-[U_*glgA*_-T_*rrnB*_-*Kan*^*R*^-D_*glgA*_]	This study
PDel2-glgABC	pDel2-[U_*glgA*_-T_*rrnB*_-*Kan*^*R*^-D_*glgC*_]	This study
pDel2-glgC	pDel2-[U_*glgC*_-T_*rrnB*_-*Amp*^*R*^-D_*glgC*_]	This study

Recombinant plasmid DNA was introduced to DH-1 by electroporation and as described before [25]. Competent DH-1 cells were prepared as follows. Cells cultured from NMS plate were harvested with sterilized, ice-cold water and centrifuged at 14,000 rpm for 2 min. Cells were washed twice and resuspended with 200 ~ 300 μL of ice-cold water. 50 μL of cell resuspension and 3 μL of plasmid DNA were mixed gently and transferred to an ice-cold 2-mm-gap cuvette. Electroporation was performed using a Gene Pulser II system (Bio-Rad, USA) at preprogrammed Ec2 setting. Immediately after electrical discharge, 1 mL of room temperature NMS medium was added to cells and transferred to 30 mL serum bottle supplied with additional 2 mL of NMS medium and 20% CH_4_. After overnight incubation in a shaking incubator, cell pellets were harvested by centrifugation and spread onto NMS plate containing 10 μg/mL of kanamycin or 25 μg/mL of ampicillin.

### Fermenter culture

Strain JHM805 was pre-cultured in a 1-L baffled flask containing 200 mL of NMS medium (standard medium with 1 g/L KNO_3_ or modified medium with 6 g/L KNO_3_) and 10 µg of kanamycin, supplied with 20% methane and 80% air. The headspace of the flask was purged at 0 h and 24 h during incubation. After incubation in a shaking incubator for 48 h, 200 mL of seed culture was transferred to the bioreactor. Bioreactor fermentation was performed in a 5-L Bioreactor (BioCNS, Daejeon, Republic of Korea) containing 3 L of NMS medium with 10 µg/ml kanamycin and 50 µM IPTG at 30 °C, with an agitation speed of 800 rpm. The gas mixture of 20% methane and 80% air, controlled by a mass flow controller (Brooks Instrument, Hatfield, PA) was supplied using microgas sparger at the rate of 320 mL/min. To maintain pH at the range of 6.9 ~ 7.1, 2 N HCl and 5 N NaOH were used.

### Analytical method

Cell growth was analyzed by measuring optical densities at 600 nm. To quantify D-LA, 150 µL of culture supernatant was collected, filtered through 0.22 µm filter, and analyzed via high performance liquid chromatography (HPLC). The separation was performed using a BioRad Aminex HPX-87H column, with 5 mM H_2_SO_4_ as the mobile phase at a 0.6 mL/min flow rate. The column temperature was maintained at 60 °C, while the refractive index (RI) detector was set to 35 °C for detection. Consumed methane concentration was analyzed using gas chromatography system (Agilent 7890B, Agilent Corporation, USA) equipped with molecular sieved 5A column and PorapakQ column at 50 °C with argon gas as a carrier gas at a constant pressure of 27 psi. The analytes were detected by thermal conductivity detector (TCD) at 250 °C.

The concentration of nitrate was analyzed by Ion chromatography system (Dionex Aquion, Thermo fischer, USA) equipped with DS6 heated conductivity cell and Dionex Ionpac AS23 RFIC column at 30 °C. 4.5 mM carbonate with 0.8 mM bicarbonate (Dionex AS23 Eluent concentrate, Thermo Fisher, USA) was used as an anion mobile phase.

## Results and discussion

### Introduction of inducible promoters for fine-tuned expression of D-LDH

In our previous study, we developed the JHM86 strain by integrating the *Lm.LDH* gene, encoding D-specific LDH (D-LDH*)*, into the chromosome of the LA-tolerant JHM80 strain and simultaneously deleting the *glgA* gene. This engineered strain, JHM86, which expresses the *Lm.LDH* gene under the control of the *glgBA* operon promoter, successfully produced 1.19 g/L of D-LA [25]. To further enhance D-LA production, we substituted the *glgBA* promoter with the more potent constitutive *mxaF* promoter. However, this approach failed to produce a viable strain, likely because of severe growth inhibition caused by D-LA toxicity. Based on a previous study [27], several promoters predicted to be weaker than *mxaF* but stronger than the *glgBA* promoter were tested to alleviate the growth defect. However, these promoters were unable to improve D-LA production (Supplementary Figure S1).

**Fig. 1:**
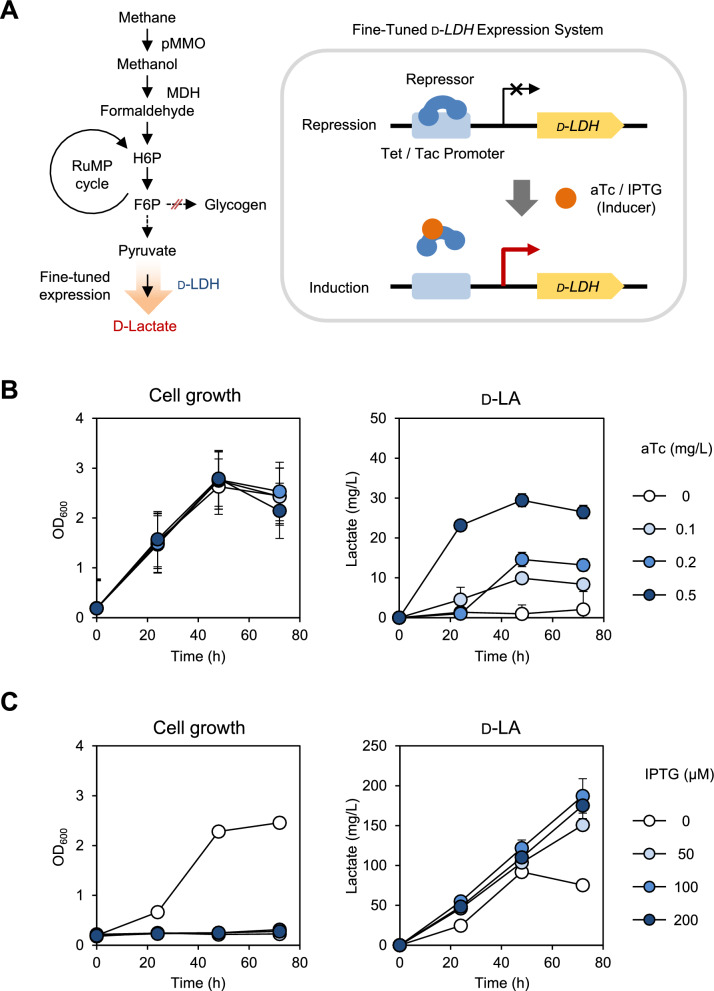
D-LA production from methane with a fine-tuned *D-LDH* expression system. **A** Metabolic pathway of D-LA production from methane and schematic overview of the fine-tuned *D-LDH* expression system, controlled by the *tet* or *tac* promoter. **B** Growth and D-LA production of JHM803, expressing *D-LDH* under the Tet promoter, in media supplemented with the indicated concentrations of aTc. **C** Growth and D-LA production of JHM804, expressing *D-LDH* under the *tac* promoter, in media supplemented with the indicated concentrations of IPTG. The initial inoculation OD_600_ was 0.2 and error bars indicate standard deviations of two independent experiments

To address this issue, inducible promoters were introduced to enable controlled expression of target genes at desired points and levels. We tested two inducible promoters to express the *D-LDH* gene: the *tet* promoter (P_*tet*_) and the *tac* promoter (P_*tac*_), which were induced by anhydrotetracycline (aTc) and Isopropyl β-D-1-thiogalactopyranoside (IPTG), respectively (Fig. 1A). The P_*tet*_ has previously been used to produce L-LA from methane in *M. buryatense*, achieving 0.8 g/L of LA in a continuous stirred bioreactor [32]*.* We inserted DNA fragments containing P_*tet*_-*LDH* and P_*tac*_-*LDH* with their repressors into the *glgA* site of JHM80 to generate JHM803 (JHM80 *ΔglgA*::P_*tet*_-*LDH*) and JHM804 (JHM80 *ΔglgA*::P_*tac*_-*LDH*), respectively.

In JHM803, D-LA production was minimal without aTc induction, demonstrating tight control of the *tet* promoter (Fig. 1B). D-LA production increased with rising concentrations of aTc, peaking at 29.5 mg/L when induced with 0.5 mg/L of aTc for 48 h (Fig. 1B). The aTc concentration used was half of the maximum tested concentration that did not cause significant growth inhibition (Supplementary Figure S2). In contrast, the *tac* promoter showed less stringent regulation than the *tet* promoter. The JHM804 strain produced up to 103.7 mg/L of D-LA at 48 h, even without IPTG induction of *D-LDH*. When 100 μM IPTG was added, D-LA production increased to 187 mg/L at 72 h, representing a 6.3-fold increase over JHM803 (Fig. 1C). Although the D-LA titers with IPTG induction were similar to those without induction during the first 48 h of cultivation (Fig. 1C), IPTG-induced cells exhibited approximately 22-fold higher D-LA production per cell OD (Supplementary Figure S3). This increase was primarily attributed to severe growth inhibition resulting from the enhanced transcription of *D-LDH*, leading to lactate toxicity under non-neutralizing conditions. Although the parental strain JHM80 can tolerate up to 8 g/L of externally added LA under neutralizing conditions [25], intracellular production of LA appears to impose greater toxicity, significantly limiting cell growth. The *tet* promoter is advantageous owing to its tight regulation. However, considering the requirement of strong *D-LDH* expression to enhance LA production, the *tac* promoter was selected for further experiments.

### Evaluation of the tac promoter for D-LA production with methane feeding

**Fig. 2:**
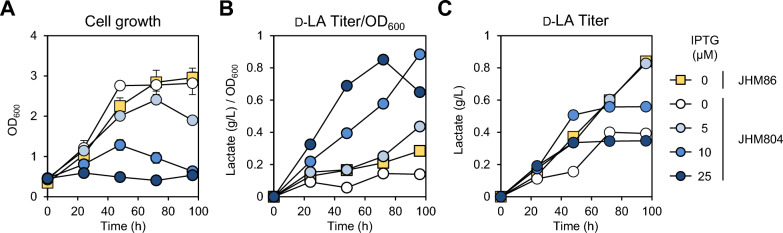
Assessment of D-LA production through regulation of *D-LDH* expression from the Tac or *glgBA* promoters. Growth (**A**) and D-LA (**C**) production of JHM86, expressing D-LDH under the *glgBA* promoter, in NMS medium, and JHM804, expressing D-LDH under the Tac promoter, in IPTG-supplemented medium. Methane was supplied every 24 h. Cell growth and D-LA production were measured during the growth. The relative D-LA content (**B**), calculated as the ratio of LA concentrations to OD_600_, was compared under different conditions. The initial inoculation OD_600_ was 0.5, and data from two independent experiments were averaged and displayed with standard deviations

We next assessed JHM804, which utilizes P_*tac*_-controlled *D*-*LDH* expression, against our previous JHM86 strain, which expresses *D*-*LDH* from the *glgBA* promoter [25]. To determine the optimal IPTG concentration for D-LA production in fed-batch culture, we cultivated JHM804 in NMS medium with various IPTG concentrations, supplying 20% methane every 24 h. Since the initial tests with 50 μM IPTG caused severe growth inhibition (Fig. 1C), we explored lower IPTG concentrations ranging from 5 to 25 μM, starting with cell inoculation at an OD_600_ of 0.5. As a control, JHM86 cells were grown in the absence of IPTG. As IPTG concentration increased, the growth of the JHM804 strain was inhibited in a concentration-dependent manner (Fig. 2A). Concurrently, the D-LA production titer per cell OD increased (Fig. 2B), reflecting the effects of lactate toxicity linked to higher *D*-*LDH* expression levels. As a result, 5 μM IPTG treatment resulted in the highest D-LA production levels, which were comparable to those observed in JHM86 (Fig. 2C). These results suggest that controlling *D-LDH* transcription with the *tac* promoter enables higher expression levels than regulation by the *glgBA* promoter at IPTG concentrations exceeding 5 μM. While LA toxicity limits D-LA production, the inducible system enables more precise control of *D-LDH* expression, potentially surpassing the production levels of JHM86 when LA toxicity is mitigated through continuous pH neutralization during bioreactor fermentation. Moreover, continuous exposure to LA stress may increase the risk of genetic mutations. Considering the observed genetic instability and limited tolerance to organic acids in *Methylomonas* sp. DH-1 [35], we concluded that constitutive D-LA production could be detrimental to cells. Consequently, maintaining a low level of LA production prior to induction may promote stable production of D-LA.

### Disruption of glucose 1-phosphate adenylyltransferase (*glgC*) to alleviate toxicity of ADP-glucose accumulation due to *glgA* deletion

**Fig. 3:**
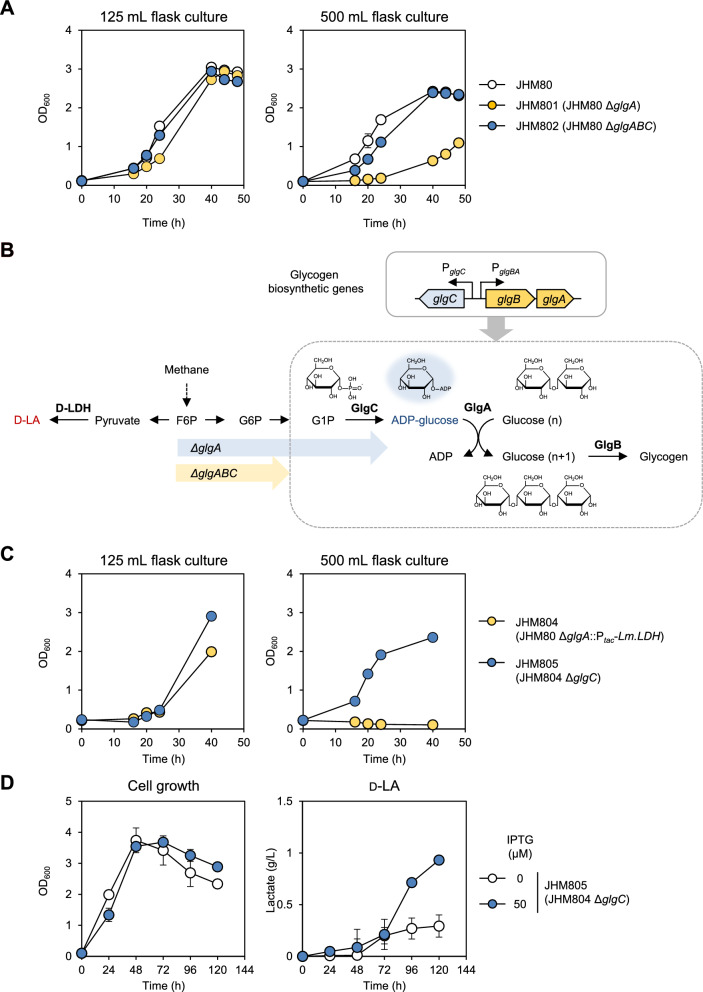
Effect of *glgC* deletion on cell growth and LA tolerance. **A** Growth comparison of indicated strains in 125 mL flasks (12.5 mL NMS medium) and 500 mL flasks (50 mL NMS medium) to examine scale-up effects. The initial inoculation OD_600_ was 0.1. **B** Metabolic pathway of glycogen synthesis from methane in *Methylomonas* sp. DH-1, highlighting the key intermediates: F6P, fructose-6-P; G6P, Glucose-6-p; G1P, Glucose-1-p. The carbon flux under carbon excess conditions is illustrated with arrows for the JHM801(*ΔglgA*) and JHM802 (*ΔglgABC*) strains, highlighting ADP-glucose accumulation resulting from *glgA* deletion. **C** Effect of *glgC* deletion on cell growth of JHM804 under different culture scales. The initial inoculation OD_600_ was 0.2 and the strains were grown in 125 mL flask containing 12.5 mL NMS medium or 500 mL flask containing 50 mL NMS medium. Each value represents the average ± standard deviation of two independent experiments. **D** Effect of IPTG addition (50 µM) on cell growth and D-LA production in JHM805 with methane supplied every 24h, with the initial inoculation OD_600_ was 0.1. Error bars indicate standard deviations of two independent experiments

Next, we attempted to scale up the culture using a bioreactor to further increase the D-LA titer. Unexpectedly, the JHM804 strain failed to grow when scaled up from a 50 mL flask to a 500 mL flask, even in the absence of the inducer IPTG. Since basal D-LA production was low without IPTG induction, LA toxicity was unlikely to be the main reason for growth inhibition. In contrast, the LA-tolerant parental strain, JHM80, showed normal growth in a 500 mL flask culture (Fig. 3A). This led us to hypothesize that the growth defects observed in larger flasks may be related to the deletion of *glgA*. Supporting this hypothesis, the previously developed JHM86 strain, which also carried a *glgA* deletion, failed to grow during scale-up in a bioreactor.

Excessive foam formation was observed in larger flasks, as shown in Supplementary Figure S4. The increased surface area, combined with a larger radius of rotation and higher angular velocity, caused more frequent collisions with the baffles in the flasks. This generated greater turbulence in the culture medium, enhancing gas transfer to the liquid phase. The improved exchange of methane and oxygen, both essential for cell growth, may have resulted in “carbon overflow,” where carbon uptake exceeded the metabolic capacity of the strain. Under such conditions, converting excess carbon into storage pathways such as glycogen serves as a natural “ameliorator” to mitigate metabolic stress [36, 37].

In *Methylomonas* sp. DH-1, along with the primary metabolism of converting methane to pyruvate, the glycogen synthesis pathway functions as a storage compound to manage excess carbon (Fig. 3B). Methane is metabolized to fructose 6-phosphate (F6P) via the ribulose monophosphate (RuMP) cycle, and F6P is then converted to glucose 1-phosphate (G1P). Glycogen synthesis involves three key enzymes: G1P adenylyltransferase (GlgC), glycogen synthase (GlgA), and glycogen branching enzyme (GlgB) (Fig. 3B). GlgC catalyzes the conversion of G1P to ADP-glucose, which is then utilized by GlgA to form linear *α*-(1 → 4)-linked glucose chains. Finally, GlgB introduces *α*-(1 → 6)-linked branches into glucose chains, resulting in a branched glycogen structure.

Therefore, with the deletion of *glgA*, the glycogen pathway halts at ADP-glucose, particularly during large-scale culture where carbon excess leads the carbon flux to glycogen accumulation (Fig. 3B). In general, the accumulation of sugar phosphates (e.g. galactose-1-phosphate, fructose-1-phosphate, and trehalose-6-phosphate) is recognized as toxic to cells—from *E. coli* to humans—due to the wasteful consumption of ATP [38]. ADP-glucose, an essential intermediate, plays a crucial role in balancing the ATP and ADP levels within cells. The adenylate energy charge (AEC), calculated as ([ATP] + 0.5 × [ADP])/([ATP] + [ADP] + [AMP]), serves as a key indicator of the cell’s metabolic state and its ability to perform energy-consuming processes. Under optimal growth conditions, the AEC is typically maintained around 0.9 [39, 40]. However, in cells accumulating ADP-glucose, the AEC has been reported to drop to 0.1 [41]. The accumulation of this intermediate has been found to be lethal in cyanobacteria, as deletion of glycogen synthase genes results in toxic ADP-glucose accumulation and cell death [41]. Interestingly, salt-induced stress has been reported to suppress this toxicity by redirecting carbon flux towards the production of osmolyte glucosylglycerol, highlighting the importance of metabolic flexibility in mitigating ADP-glucose accumulation. Despite its significance, comprehensive studies on this intermediate remain limited.

To test the effect of ADP-glucose accumulation, we deleted either the *glgA* gene alone or the entire glycogen synthesis operon (*glgA*, *glgB*, and *glgC*) in JHM80 and evaluated their growth in a 500 mL flask (Fig. 3A). In a 500 mL flask, the JHM801 strain (JHM80 *ΔglgA*) exhibited significant growth inhibition, whereas the JHM802 strain (JHM80 Δ*glgABC*) showed restored growth. The growth defect in the JHM801 strain was also evident in the smaller 125 mL flask culture, although it was less pronounced than in the 500 mL setup. The observation that deleting *glgA* has a greater negative impact on cell growth as culture volume increases suggests that during scale-up, metabolic flux towards ADP-glucose may intensify due to the redirected carbon flux towards glycogen synthesis in a carbon-rich environment. To address this issue, we alleviated ADP-glucose accumulation in the JHM804 strain by deleting the *glgC* gene. As expected, the resulting strain, JHM805 (JHM804 Δ*glgC*), exhibited growth recovery in both 125 mL and 500 mL flask cultures (Fig. 3C). We also evaluated the capacity of JHM805 to produce D-LA. While *D-LDH* induction with 50 μM IPTG severely inhibited the cell growth of JHM804 (Fig. 1C), JHM805 exhibited almost normal growth and produced up to 0.93 g/L of D-LA (Fig. 3D). These results indicate that deleting *glgC* not only facilitates culture scale-up but also potentially increases LA tolerance by improving overall cell fitness. Based on our observations, *glgC* deletion did not significantly impair cell growth or biomass yield, suggesting that metabolic burden was minimal under our experimental conditions. Alternative strategies to mitigate ADP-glucose toxicity, such as inducing salt stress [41] or modifying glycogen metabolism via ADP-glucose-involved genes [42], could also be considered. However, due to the limited knowledge of the *Methylomonas* sp. DH-1 strain and its expected instability under external stress, we concluded that *glgC* deletion is the most suitable approach in this context.

In our initial strain design for D-LA production, we deleted *glgA* gene to prevent competitive use of a carbon source for glycogen synthesis [25]. Notably, certain methanotrophic bacteria can accumulate substantial glycogen levels up to 30% of the dry cell weight (DCW) [43, 44]. Thus, inhibition of glycogen synthesis is an important strategy for increasing cellular lipid or protein levels [20, 42]. However, deleting the *glgC* gene might be a more effective strategy. This approach not only halts glycogen production but also reduces the accumulation of potentially toxic ADP-glucose. However, considering that *M. buryatense* exhibits only a minor growth defect during flask or bioreactor cultivation following *glgA* deletion [8, 20], further studies are needed to elucidate the underlying mechanisms of carbon storage. Interestingly, *M. buryatense* contains annotated genes for both glycogen and sucrose biosynthesis pathways, whereas *Methylomonas* sp. DH-1 strain has genes exclusively annotated for glycogen, indicating a potential difference in carbon storage preference. Further investigation into the regulation of carbon storage biosynthesis is necessary to optimize the carbon excess conditions and enhance the production of various chemicals in methanotrophs.

### Bioreactor fermentation for D-LA production

**Fig. 4:**
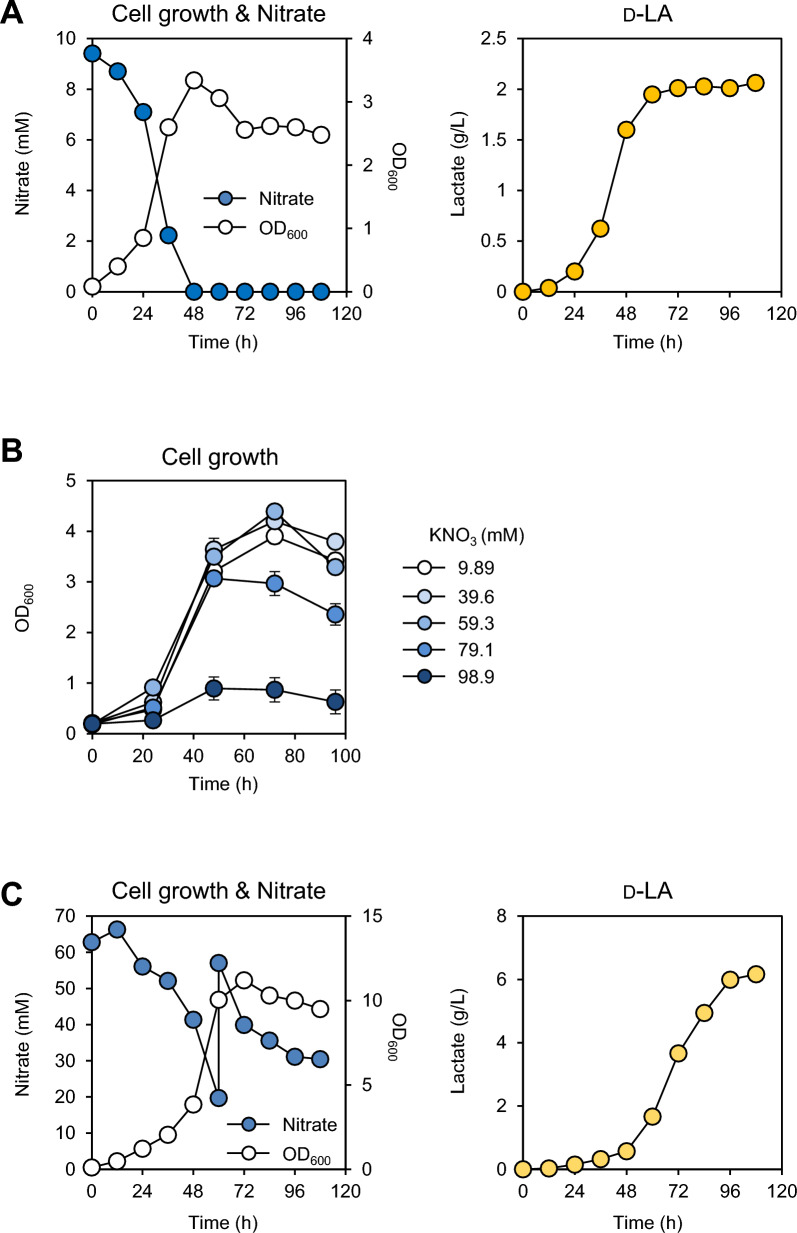
Batch fermentation and nitrate feeding fermentation of JHM805 strain in continuous stirred bioreactor. **A** Growth and D-LA production of JHM805 strain in a 5L bioreactor containing 3 L NMS medium, supplemented with 10 μg/mL kanamycin and 50 μM of IPTG. A gas mixture of 20% (v/v) of methane and 80% (v/v) of air was continuously supplied and the initial inoculation OD_600_ was 0.1. Nitrate depletion coincides with the onset of D-LA production, indicating a shift in metabolic flux upon nitrogen limitation. **B** Growth of JHM805 in NMS media containing various concentrations of KNO_3_ to evaluate the effect of nitrate availability. The initial inoculation OD_600_ was 0.2 and error bars indicate the standard deviation of two independent experiments. **C** JHM805 fermentation in a 5-L bioreactor with modified NMS medium (6 × KNO_3_) with 10 μg/mL kanamycin and 50 μM of IPTG. Nitrate was replenished before depletion to prevent nitrogen limitation and 20% (v/v) of methane and 80% (v/v) of air were continuously supplied and the initial inoculation OD_600_ was 0.1. Cell growth, D-LA production, and nitrate consumption were monitored during the growth

The final strain, JHM805, was cultured in a 5-L bioreactor filled with 3 L of NMS medium supplemented with 50 µM IPTG. A mixture of 20% (v/v) methane and 80% air (v/v) was supplied continuously. Throughout cultivation, cell growth, D-LA production, and KNO_3_ concentrations were monitored. The JHM805 strain exhibited exponential growth until nitrate depletion occurred at 48 h (Fig. 4A). D-LA production increased with cell growth but significantly decreased after nitrate depletion, nearly halting 24 h post-depletion. After 108 h, the D-LA production reached 2.06 g/L (Fig. 4A).

Nitrogen supplementation has been shown to enhance the production of various metabolites, including LA and lipids, in different organisms [45, 46]. In *Rhizopus arrhizus*, maintaining a low C/N ratio through NH_4_NO_3_ supplementation significantly increased LA production [47]. Additionally, nitrogen limitation leads to a high C/N ratio, which often triggers the redirection of carbon flux toward storage compound synthesis rather than product formation [48]. The beneficial effects of nitrogen supplementation are primarily attributed to increased biomass formation and enhanced metabolic activity, as nitrogen is a fundamental component of amino acids, nucleotides, and cofactors essential for cell growth and enzyme production [49]. Particularly in gas-converting microbial processes, increasing biomass is more critical than in conventional fermentations using soluble substrates, considering the challenges of achieving high cell density in gas-fermentation [50]. In methanotrophs, studies have used higher nitrate concentrations than those in standard NMS medium to enhance target material production. In *M. capsulatus* Bath, 3 × nitrate (3.0 g/L KNO_3_) was used for bioreactor operation to increase mevalonate production [50]. Additionally, 4 × nitrate supplementation has been used to enhance lipid production [20], while 8 × nitrate supplementation has been applied to achieve higher lactate production with increased cell growth [32] in *M. buryatense*. As in conventional fermentations using soluble substrates, increasing biomass production by supplying a higher nitrogen source may also be an effective strategy for enhancing target compound production in methanotroph cultures.

Based on these findings, we decided to improve cell growth and D-LA production by supplying more nitrate as an N source. To assess the tolerance of JHM805 to elevated nitrogen levels, we evaluated its growth at 4x, 6x, 8x, and 10x  KNO_3_ concentrations relative to the standard NMS medium (where 1 g/L KNO_3_ is equivalent to 9.89 mM). Growth remained unaffected at concentrations up to 6 g/L KNO_3_ (59.3 mM), indicating that nitrate supplementation does not impose significant inhibitory effects (Fig. 4B).

Consequently, bioreactor cultivation was conducted using 6 g/L KNO_3_ in the NMS medium. During cell culture, nitrate was added once at a concentration below the inhibitory level, before complete depletion occurred. This approach improved cell growth compared with batch cultivation using 1 g/L KNO_3_. In the fed-batch culture, the maximum OD_600_ reached 11.2, which is a 3.35-fold increase compared to the OD_600_ of 3.34 observed in the batch culture using 1 g/L KNO_3_ (see Fig. 4A). The D-LA titer reached 6.17 g/L after 108 h of fermentation (Fig. 4C), which is the highest reported among studies on D- or L-LA production in microorganisms using methane or methanol as carbon sources (Table 3). The D-LA titer (g/L) per OD_600_ was 0.65, which was lower than the 0.83 observed in the bioreactor culture without nitrate supplementation (Fig. 4A and Supplementary Table S1). However, in the modified NMS medium, D-LA productivity reached 0.057 g/L/h. Based on these data, higher cell growth due to increased nitrogen supply likely led to the observed increase in lactate titer and productivity. A higher or additional nitrate supply is unlikely to have shifted the intracellular metabolic flux toward lactate production, given the decreased D-LA titer (g/L) per OD_600_. The D-LA productivity of 0.057 g/L/h is not only the highest rate ever reported in methanotrophs but also represents a 7.12-fold increase from the 0.008 g/L/h achieved in our previous study [25] (Fig. 4C).

**Table 3:** Production of LA and other organic acids from methane

Host strain	Products	Strategies	Culture	Carbon source, Titer	References
*Production of LA*					
*Methylomonas* sp. DH-1	D-LA	1. Adaptive evolution to increase lactate tolerance 2. Introducing *D-LDH* from *L. mesenteroides*	Fed-batch (144 h)	Methane, 1.19 g/L	[25]
*Methylomonas* sp. DH-1	D-LA	1. Introducing inducible promoter for the expression of *D-LDH* from *L. mesenteroides* 2. Elimination of ADP-glucose accumulation 3. Optimizing fermentation conditions for large-scale culture 4. Prevent nitrogen deficiency via nitrate supplementation	Fed-batch (108 h)	Methane, 6.17 g/L	This study
*Methylomicrobium buryatense* 5GB1S	L-LA	1. Introducing of *L-LDH* from *L. helveticus*	Constant feeding (96 h)	Methane, 0.8 g/L	[32]
*Methylomicrobium buryatense* 5GB1	L-LA	1. Introducing *L-LDH* from *L. helveticus* 2.Optimizing fermentation conditions	Batch (96 h)	Methane, 0.5 g/L	[33]
*Production of other organic acids*					
*Methylomonas* sp. DH-1	Succinate	1. Knock-out of succinate dehydrogenase 2. Overexpression of glyoxylate shunt 3. Disruption of pyruvate formate lyase and acetate kinase phosphotransacetylase	Fed-batch (120 h)	Methane, 195 mg/L	[26]
*Methylosinus trichosporium* OB3b	3-Hydroxypropionic acid	1. Overexpression of precursor supply genes 2. Improving the supply of redox cofactor	Constant feeding (42 h)	Methane, 60.59 mg/L	[31]
*Methylosinus trichosporium* OB3b	4-Hydroxybutyrate	1. Introducing inducible promoter for the expression of succinate semialdehyde dehydrogenase from *P. gingivalis* and NADPH-dependent succinate semialdehyde reductase from *E. coli*	Fed-batch (144 h)	Methane, 10.5 mg/L	[55]

While our study successfully improved D-LA production in *Methylomonas* sp. DH-1, further optimization strategies can enhance its industrial applicability. A key challenge in D-LA production by this strain is its low tolerance to organic acids, making it essential to explore rational or random metabolic engineering approaches to improve toxicity resistance. Notably, we identified small peptides that are predicted to modulate the substrate specificity of efflux pumps, potentially enhancing acetate export [35]. Engineering these efflux pumps to efficiently remove intracellularly accumulated D-LA could be a promising strategy to further improve production.

Additionally, while we optimized nitrate supplementation for bioreactor cultivation, previous studies suggest that modifying the metal ion composition in the culture medium [51] or supplementing other components (e.g., phosphate and organic acids) [52, 53] could enhance methane consumption and cell growth of methanotrophs. Given that genetic and metabolic engineering of methanotrophs is still in its early stages, many unexplored opportunities remain to further enhance their performance. These findings highlight the potential of methanotroph-based bioproduction of high-value chemicals and provide a strong foundation for future research.

## Conclusions

The bioconversion of methane, an abundant carbon source, into D-LA, a biodegradable plastic monomer, represents a promising strategy. Methane-based fermentation offers economic advantages by using a low-cost, widely available feedstock, thereby reducing production costs. In this study, we engineered an LA-tolerant strain of *Methylomonas* sp. DH-1 to improve LA production by focusing on three critical aspects. First, the use of an inducible promoter for *D-LDH* expression effectively reduced lactate toxicity, a major challenge in improving production. Second, we addressed the metabolic imbalance during scale-up, particularly ADP-glucose accumulation due to *glgA* deletion (encoding glycogen synthase). This issue was resolved by deleting the *glgC* gene, which encodes glucose-1-phosphate (G1P) adenylyltransferase. Finally, this study contributes to the limited research on large-scale production systems utilizing methanotrophs, demonstrating *Methylomonas* sp. DH-1 as a promising platform for sustainable D-LA production from methane. Our optimized strain achieved a final titer of 6.17 g/L in a 5-L fed-batch fermenter by preventing nitrogen source depletion, marking the highest D-LA titer reported in methanotrophs to date.

## Supplementary Information

Additional file 1.

## Data Availability

All data generated or analyzed during this study are included in this published article and its supplementary information files.
