# Supplementary material for: Enhancing D-lactic acid production from methane through metabolic engineering of Methylomonas sp. DH-1
Source: Microb Cell Fact. 2025 Mar 25;24:70. doi: 10.1186/s12934-025-02695-z (PMC11934524; doi:10.1186/s12934-025-02695-z)
Supplement: Supplementary file 1 — Additional file 1. [file 12934_2025_2695_MOESM1_ESM.pdf]

Supplementary data

Enhancing D-lactic acid production from methane through metabolic engineering of *Methylomonas* sp. DH-1

Supplementary Table

Table S1. D-LA production titer per cell mass depending on strains and culture conditions

Strain	Culture	Culture time (h)	IPTG (μM)	D-LA Titer (g/L) /OD_{600}
JHM86	Flask fed-batch	96	0	0.28
JHM804	Flask fed-batch	96	0	0.13
		96	5	0.44
		96	10	0.88
		85	25	0.85
JHM805	Flask fed-batch	120	50	0.32
	Bioreactor (w/o nitrate feeding)	108	50	0.83
	Bioreactor (w/ nitrate feeding)	108	50	0.65

Supplementary Figures

A

Gene	Definition	Relative strength
AYM39_19855	50S ribosomal protein L31	0.07
glgA	Glycogen synthase	1
AYM39_17865	Elongation factor Tu	1.5
AYM39_02490	Transaldolase	5.1
AYM39_15615	Methanol dehydrogenase	54.9

B

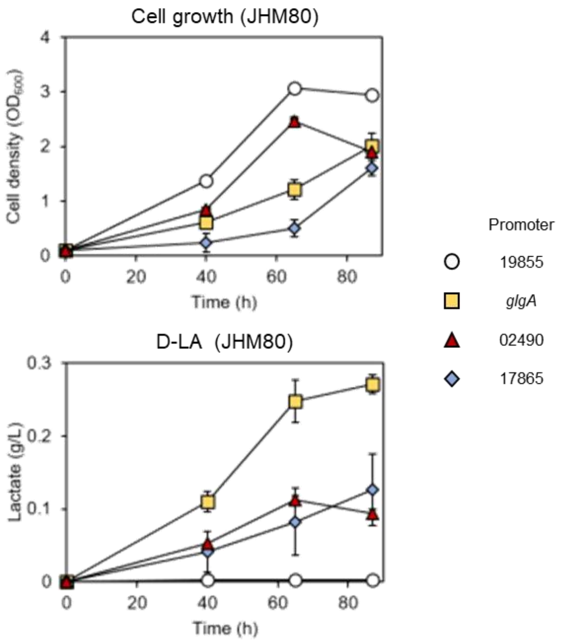

Figure S1. Effect of promoter strength of *LDH* expression integrated in JHM80 strain. **A.** Relative strength of five different constitutive promoters tested, based on the previously published data (1). **B.** Cell growth and LA production of JHM80 strains with LDH integrated under the indicated promoters. Error bars represent the standard deviations of two independent experiments.

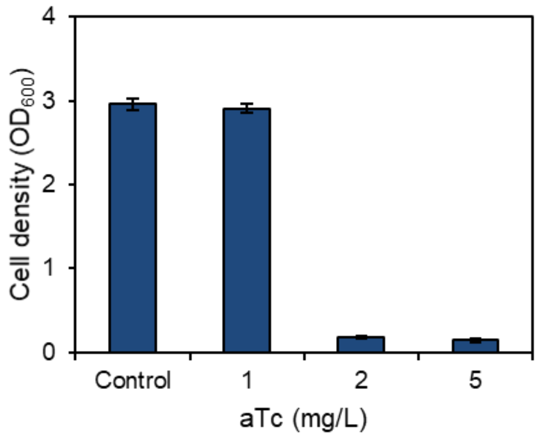

Figure S2. Effect of anhydrotetracyclin (aTC) addition on cell growth in the JHM80 strain. Cells were grown in the absence or presence of aTC for 40 h. Error bars indicate standard deviations of two independent experiments.

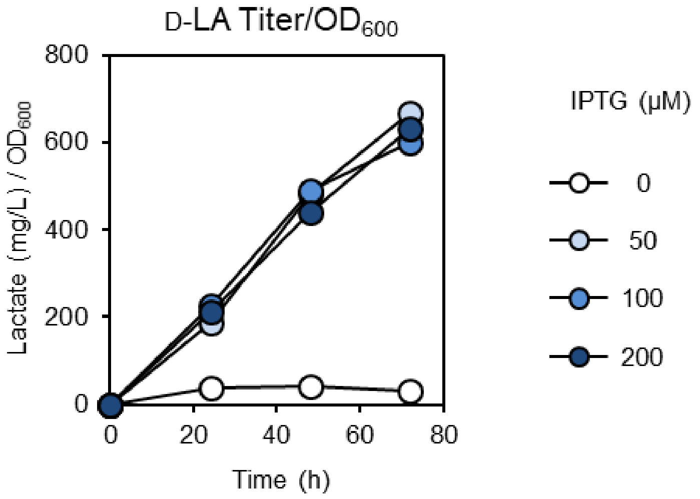

Figure S3. The relative amount of D-LA titer, calculated as the ratio of LA concentrations to OD_{600} values, based on the data shown in Figure 1C.

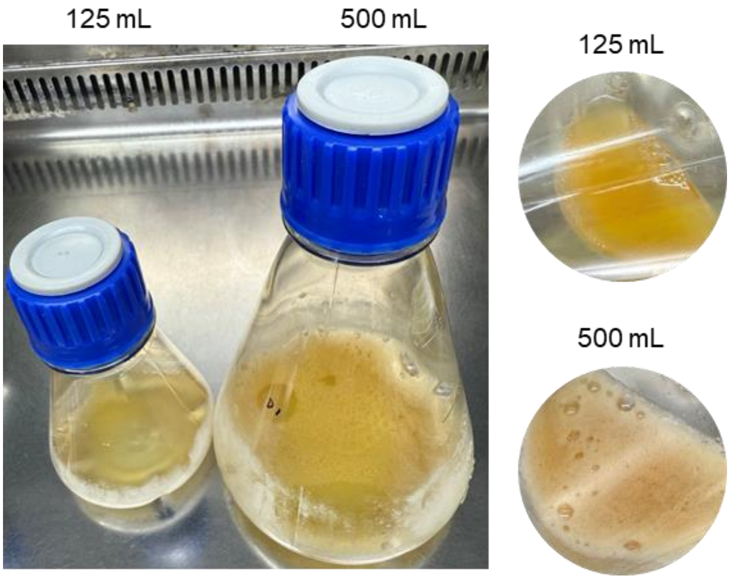

Figure S4. Effect of scale-up flask size on foam formation. Photographs of each flasks are shown together (left) and in closed-up (right). *Methylomonas* sp. DH-1 cells were grown in 125 mL or 500 mL flasks containing 12.5 mL or 50 mL of NMS media, respectively.

Supplementary References

1. Lee HM, Ren J, Yu MS, Kim H, Kim WY, Shen J, et al. Construction of a tunable promoter library to optimize gene expression in Methylomonas sp. DH-1, a methanotroph, and its application to cadaverine production. Biotechnol Biofuels. 2021;14(1):228.
